# Optimizing phenotypic data analysis strategies to enhance genomic prediction in winter wheat breeding

**DOI:** 10.3389/fpls.2026.1898203

**Published:** 2026-08-04

**Authors:** Ravindra Reddy Gundala, Georg Witte, Jost Doernte, Michael Koch, Yusheng Zhao, Jochen Christoph Reif

**Affiliations:** 1 Leibniz Institute for Plant Genetics and Crop Plant Research, Seeland, Germany; 2 Deutsche Saatveredelung AG, Lippstadt, Germany

**Keywords:** breeding stage, genomic prediction, mixed linear model (MLM), phenotypic data analysis strategies, plant breeding, quantitative genetics, two-stage analysis, winter wheat (*Triticum aestivum* L)

## Abstract

Proper analysis of phenotypic data is essential for reliable genomic prediction (GP) and sustained genetic gain in breeding programs. In this study, we used phenotypic and genotypic data generated from the winter wheat breeding program of Deutsche Saatveredelung AG (DSV), Lippstadt, Germany, comprising 1,941 genotypes and 6,335 SNP markers across three traits: grain yield, plant height, and heading date. We evaluated the impact of different two-stage analysis strategies: one using the environment (year × location combination) as the analysis unit (TS-S1) and the other using the breeding stage as the analysis unit (TS-S2), each with and without accounting for breeding-stage effects (-YesBS and -NoBS), on the computation of best linear unbiased estimates (BLUEs) and genome-wide prediction ability (PA), defined as the correlation between BLUEs and predicted values. The performance of these 4 different second stage models (TS-S1-NoBS, TS-S1-YesBS, TS-S2-NoBS, and TS-S2-YesBS) were evaluated using the extended genomic best linear prediction (EGBLUP) model under 5-fold cross validation (5-fold CV), leave one year out cross validation (LOY-CV) and leave one breeding stage out cross validation (LOBS-CV) scenarios. In the presence of the strong breeding stage effect ignoring the breeding stage in the model resulted in biased BLUEs and overestimated prediction abilities in the 5-fold CV, and underestimated prediction abilities in the LOY-CV and LOBS-CV. These unstable prediction abilities are driven by confounded environmental effects in the BLUEs due to omission of the breeding-stage effect. In contrast, models that accounted for the breeding stage produced more reliable BLUEs and more stable prediction results across all cross-validation scenarios, with TS-S1-YesBS performing best overall. Overall, our findings demonstrate that breeding stage effects mainly arise from differences in growing conditions and must be adequately considered. Therefore, including breeding stage in phenotypic models is critical to obtaining unbiased BLUEs and ensuring accurate genomic prediction and selection decisions.

## 1 Introduction

Feeding a growing global population requires a rapidly increasing food production. Wheat is one of the main staple crops and plays a key role in global food security. Thus, enhancing the rate of genetic gain in wheat is crucial to meeting future demand (Ray et al., 2013). In this context, genomic prediction (GP) (Meuwissen et al., 2001) offers a strong opportunity to increase genetic gain per unit time (Voss-Fels et al., 2019). Genomic prediction uses a comprehensive training set containing both phenotypic and genotypic data to predict the performance of individuals that have only been genotyped, not phenotyped. Prediction ability (PA), defined as the correlation between observed and predicted values, is a key factor that describes the success of genomic prediction. Prediction ability depends on the complex interplay among several factors, including trait heritability, genetic architecture, genetic diversity, population structure, marker density, the genetic relationship between the training and test populations, genotype by environment interaction, training population size, and phenotypic data quality (Combs and Bernardo, 2013; Lian et al., 2014; He et al., 2016; Werner et al., 2020; Zhao et al., 2021; Alemu et al., 2024). A large training set with high-quality phenotypic data is especially important (Akdemir and Isidro-Sánchez, 2019; Zhao et al., 2021; Thomsen et al., 2026). Thus, precise best linear unbiased estimates (BLUEs) commonly obtained in linear mixed models are crucial for the success of genomic prediction.

A breeding cycle in a plant breeding program begins with crossing parental lines and ends with the release of a new variety following a series of yield evaluations. Typically, three stages of yield trials are conducted prior of entering the wheat registration trials (WP): preliminary yield trials (WP-3), advanced yield trials 1 (WP-2), and advanced yield trials 2 (WP-1). In the preliminary yield trials (WP-3), a large number of candidate lines are evaluated at a limited number of locations, often without replication. As selection progresses through WP-2 and WP-1, the number of candidate lines decreases, while the number of testing environments and the level of replication increase to obtain more precise estimates of line performance. Hereafter, these three yield trial phases (WP-3, WP-2, and WP-1) are collectively referred to as breeding stages.

In practice, breeding companies evaluate trials from multiple breeding stages and different breeding cycles within the same year. Consequently, the resulting datasets comprise heterogeneous multi-environment trials that are typically analysed using linear mixed models. A single-stage model provides BLUEs for fixed effects and best linear unbiased predictions (BLUPs) for random effects while accounting for the variance–covariance structure of all observations. This approach is considered the gold standard (Smith et al., 2001; Welham et al., 2010; Piepho et al., 2012). However, due to the heterogeneous nature of breeding trials and the limited connections between them, single-stage analysis can be complex, may have convergence issues, and may not adequately accommodate all sources of variation. Therefore, two-stage analysis is often a simpler, more practical alternative (Möhring and Piepho, 2009).

In the standard two-stage approach, each environment (defined as a year × location combination) is usually analysed separately in the first stage to account for the trial design and within-environment error. Then, a second-stage model accounts for environmental effects. Genomic prediction can then be applied in a third stage by combining the BLUEs from the second stage with genotypic data (Piepho et al., 2012). The success of this approach depends critically on the accuracy of the BLUEs. If the first- and second-stage mixed models fail to adequately account for environmental and design effects, residual non-genetic variation may remain in the BLUEs. Such bias can propagate into the genomic prediction model, influencing the prediction ability and ultimately leading to less efficient selection decisions.

Previous studies suggest that simple linear mixed models that account for row–column effects or one-dimensional blocking are often sufficient to capture field heterogeneity in the first stage, rather than more complex spatial models (Bernal-Vasquez et al., 2014). In addition, when years are poorly connected, for example with only one check genotype, fitting year as a fixed effect in the genomic prediction stage can improve correction for year effects (Bernal-Vasquez et al., 2014).

Nevertheless, within a single environment, data from multiple breeding stages (WP-3, WP-2, and WP-1) originating from different breeding cycles are often available and are typically only weakly connected. Furthermore, trials conducted within different breeding stages in the same environment are usually grown in separate fields, which may introduce additional environmental heterogeneity and further weaken the connections among stages. Under these conditions, it is unclear whether the standard two-stage approach provides precise BLUEs when breeding stage effects are not included. Therefore, this study evaluated whether including breeding stage improves the computation of BLUEs and ultimately also the accuracy of genomic prediction. In addition, we evaluated a further two-stage analysis in which the unit of analysis is the breeding stage within a year rather than the environment. This approach allows genotype × location interaction to be accounted for in the first stage for advanced breeding stages (WP-1 and WP-2). In the second stage, across-year means are estimated either by accounting only for the year effect or by including both year and breeding stage effects. The objectives of this study were: (i) to assess different two-stage analyses for unbiased estimation of BLUEs; (ii) to evaluate their impact on predicting genotype performance within a year across traits; (iii) to evaluate their impact on predicting genotype performance within a breeding stage across traits; and (iv) to propose a suitable phenotypic analysis strategy for efficient genomic prediction when breeding stages are poorly connected.

## 2 Materials and methods

### 2.1 Plant material, genotyping and field trials

The genotypic and phenotypic data used in this study originated from the winter wheat breeding program of the Deutsche Saatveredelung AG (DSV), Lippstadt, Germany (Gundala et al., 2025) (**Supplementary Table 1**). Phenotypic evaluations were conducted between 2020 and 2023 across 23 locations in Germany (18), Poland (2), the Czech Republic (2), and Austria (1), covering approximately 41,000 plots and 3972 genotypes over four years (**Supplementary Table 1**). The trials encompassed three breeding stages—WP-3, WP-2, and WP-1—which represent the number of years before genotypes enter the official variety registration process. The corresponding evaluated generations were F_4:5_ for WP-3, F_4:6_ for WP-2, and F_4:7_ for WP-1.

Three traits were recorded: grain yield (plot yield at 14% relative grain moisture content extrapolated to dt ha^-1^), plant height (cm from soil surface to spike tips), and heading date (days from January 1 to BBCH 59). Each year, up to 20 locations were used. An alpha-lattice design with two replications was used for WP-1 and WP-2, while WP-3 employed an unreplicated standard design with checks. Furthermore, the number of genotypes evaluated at each breeding stage varied across years (2020–2023), ranging from 716 to 737 in WP-3, 179 to 223 in WP-2, and 69 in WP-1 (**Supplementary Table 1**). The field trials were split into trials connected through up to five released varieties. Plot sizes ranged from 5.25 to 18 m².

### 2.2 Genotypic data analysis

Genotyping was performed using the Illumina Infinium 7K and 25K SNP arrays (TraitGenetics, Gatersleben, Germany), which were derived from the publicly available 90K SNP array (Wang et al., 2014). The number of genotypes assayed with each SNP array across years is provided in **Supplementary Table 2**. The genotyping was conducted in batches over the years 2020-2023. After merging SNP data, genotypes without corresponding phenotypic data were excluded (**Supplementary Table 1**). Markers with a minor allele frequency (MAF) below 5% and more than 80% missing values were filtered out, resulting in a refined dataset of 6,335 SNP markers across 1,941 genotypes. Because the 7K and 25K chips were derived from the same 90K platform, a substantial overlap of markers was present, leading to 6,335 high-quality shared SNPs after filtering. Missing values were imputed using marker means. The Rogers’ distance was calculated among all genotypes and a principal coordinate analysis (PCoA) (Gower, 1966) was performed using the “cmdscale” function of the R package “stats” (R Core Team, 2021). In addition, another non-parametric population structure analysis was conducted using the R package “AWclust” (Gao and Starmer, 2010). “AWclust” estimates allele sharing distance (ASD) (Bowcock et al., 1994; Mountain and Cavalli-Sforza, 1997) among individuals and visualizes genetic relationships using multidimensional scaling (MDS) (Borg, 2025). Unlike model-based approaches, non-parametric approaches does not rely on Hardy-Weinberg equilibrium assumptions, making it particularly suitable for breeding populations, where these assumptions are frequently violated because of artificial selection, relatedness, and non-random mating (Alhusain and Hafez, 2018; Bernardo, 2020).

### 2.3 Phenotypic data analysis

The experimental designs differed across environments, and a fully consistent weighted analysis across all traits and analysis strategies was not feasible. Therefore, two unweighted two-stage analysis strategies were applied, each with two variants in the second stage, to assess the impact of phenotypic analysis methods on genomic prediction ability.

In the strategy 1, BLUEs were computed separately for each environment, defined as the unique combination of year and location, using the following linear mixed model as baseline model Equation 1:

(1) $$y_{ijkl}\;∼\;\mu +\;g_{i}\;+t_{j}\;+r_{jk}\;+\;\;b_{jkl}\;+\;\epsilon_{ijkl},$$

where $y_{ijkl}\;$is the plot level trait measurement, μ is the mean, $g_{i}$ is the effect of $i^{th}$ genotype, $t_{j}$ is the $j^{th}$ trial effect, $r_{jk}$ refers to the effect of $k^{th}$ replication in $j^{th}$ trial, $b_{jkl}$ indicates the effect of $l^{th}$ block in the $k^{th}$ replication in the $j^{th}$ trial, and ϵ relates to the residuals.

Based on the within-environment BLUEs $\left(y_{ij}\right)$, across-environment BLUEs were subsequently estimated using following baseline model Equation 2:

(2) $$y_{ij}\;∼\;\mu +\;g_{i}\;+\;e_{j}\;+\;\epsilon_{ij},$$

where μ is the mean, $g_{i}$ is the effect of $i^{th}$ genotype, $e_{j}$ refers to the effect of the j^th^ environment.

In strategy 1, two variants were considered in the second stage. In the first variant, the environment was defined as year × location in model (Equation 2) and is referred to as TS-S1-NoBS (standard second stage model). In the second variant, the environment was defined as year × location × breeding stage in model (Equation 2) and is referred to as TS-S1-YesBS.

In the strategy 2, a breeding stage within each year was taken as the analysis unit. Within each year, BLUEs within each breeding trial stage were calculated using the following linear mixed model as baseline model Equation 3:

(3) $$y_{ijklmn}\;∼\;\mu +\;g_{i}\;+l_{j}+\;{\left(gl\right)}_{ij}\;+\;t_{jk}+r_{jkl}\;+\;b_{jklm}+\;\epsilon_{ijklmn},$$

where $y_{ijklmn}\;$is the plot level trait measurement, μ is the mean, $g_{i}$ is the effect of $i^{th}$ genotype, $l_{j}$ is the $j^{th}$ location effect, $\;{\left(gl\right)}_{ij}$ is the genotype by location effect, $t_{jk}$ is the $k^{th}$ trial effect in $j^{th}$ location, $r_{jkl}$ refers to the effect of $l^{th}$ replication in the $k^{th}$ trial in the $j^{th}$ location, $b_{jklm}$ indicates the effect of $m^{th}$ block in the $l^{th}$ replication in the $k^{th}$ trial in the $j^{th}$ location, and ϵ relates to the residuals.

Based on BLUEs within each breeding trial stage $\left(y_{ijk}\right)$, BLUEs across years were calculated using following baseline model Equation 4:

(4) $$y_{ijk}\;∼\;\mu +\;g_{i}\;+\;e_{j}\;+\;s_{jk}+\;\epsilon_{ijk},$$

where μ is the mean, $g_{i}$ is the effect of $i^{th}$ genotype, $e_{j}$ is the effect $j^{th}$ year, $s_{jk}$ refers to the $k^{th}$ breeding stage effect in the $j^{th}$ year and ϵ relates to the residuals. In strategy 2, two variants were also considered. In the first variant, the breeding stage effect ( $s_{jk}$) in model (Equation 4) was omitted (TS-S2-NoBS). In the second variant, the breeding stage effect ( $s_{jk}$) in model (Equation 4) was included and is referred to as TS-S2-YesBS.

In all the above models, the genotypic effect was modelled as a fixed effect and BLUEs were computed. All the other effects were fitted random. Outlier correction was performed using method 4 “Bonferroni-Holm with re-scaled median absolute deviation standardized residuals” as described in literature (Bernal-Vasquez et al., 2016). By extracting the variance components by fitting all the effects in the above models as random effects the repeatabilities ( $r^{2}$) and heritabilities ( $H^{2}$) were estimated using the following models (Equations 5–7). The environments that had an average correlation of less than 0.1 with the other environments and/or a repeatability less than 0.3 were removed (Lell et al., 2025).

(5) $$r^{2}=\frac{\sigma_{g}^{2}}{\sigma_{g}^{2}+\frac{\sigma_{\epsilon }^{2}}{n}},$$

(6) $$H^{2}=\sigma_{g}^{2}/(\sigma_{g}^{2}+\frac{\sigma_{\epsilon }^{2}}{N_{env}}),$$

(and)

(7) $$H^{2}=\sigma_{g}^{2}/(\sigma_{g}^{2}+\frac{\sigma_{gl}^{2}}{N_{l}}\;+\;\frac{\sigma_{\epsilon }^{2}}{N_{l}\times N_{r}}),$$

where $\sigma_{g}^{2}$ is the estimated genotypic variance, $\sigma_{\epsilon }^{2}$ is the estimated residual variance, n is the average number of replications per genotype, $N_{env}$ the average number of environments a genotype was measured in, $\sigma_{gl}^{2}$ is the estimated variance due to genotype by location interaction, $N_{l}$ is the average number of locations per genotype, and $N_{r}$ is the average number of times a genotype replicated per location. All the calculations were made using the R Statistical Software (R Core Team, 2021) and the “AsReml” package (David, 2019).

### 2.4 Genomic prediction model and validation schemes

Varietal selection is ultimately based on overall genetic merit, which includes both additive and non-additive genetic effects. Therefore, we used EGBLUP (extended genomic best linear unbiased prediction) (Jiang and Reif, 2015) considering both additive and epistasis effects was used as the primary genomic prediction model Equation 8 throughout the study:

(8) $$y=µ+g_{A}+g_{E}+\epsilon$$

where y represents the vector of genotype BLUEs averaged across environments or years. The model includes an intercept µ, a vector of additive genetic effects $g_{A}$, a vector of additive × additive epistatic effects $g_{E}$, and a residual vector ϵ. The additive genetic effects $g_{A}=(g_{A1},\;g_{A2},\ldots ,g_{An})$ are assumed to follow a normal distribution: $g_{A}∼N(0,G_{A}\sigma_{A}^{2})$, where $\sigma_{A}^{2}$ denotes the additive genetic variance. The additive genetic relationship matrix $G_{A}$ was computed using the “first method” described by (VanRaden, 2008). Similarly, the epistatic effects $g_{E}=(g_{E1},\;g_{E2},\ldots ,g_{En})$ follow $g_{E}∼N(0,G_{E}\sigma_{E}^{2})$, where $\sigma_{E}^{2}$ is the additive × additive epistatic variance. The epistatic relationship matrix $G_{E}$ was derived as the Hadamard product of the additive relationship matrix $G_{A}$ (Henderson, 1985; Jiang and Reif, 2015). Genomic predictions were performed using the BGLR package (Pérez and de los Campos, 2014).

We evaluated prediction ability using five-fold cross-validation (5-fold CV), leave-one-year-out cross-validation (LOY-CV), and leave-one-breeding stage-out cross-validation (LOBS-CV). For the 5-fold CV, the dataset was randomly partitioned into five equally sized subsets (folds). In each iteration, one-fold was used as the test set while the remaining four folds served as the training set, ensuring that each fold was used once as the test set. Prediction ability was calculated as the correlation between predicted and observed values. This procedure was repeated for 50 independent random partitions, and the mean prediction ability across all runs was reported (**Supplementary Figure 1a**).

For the LOY-CV, data from one year was used as the test set while data from all remaining years were used as the training set. This process was repeated until each year had served once as the test set. Prediction ability was calculated for each test year and reported accordingly (**Supplementary Figure 1b**). Similarly, for the LOBS-CV, data from one breeding stage was used as the test set while data from all the remaining breeding stages were used as the training set. This process was repeated until each breeding stage had served once as the test set. Prediction ability was calculated for each test breeding stage and reported accordingly.

## 3 Results

### 3.1 Absence of underlying population structure

**Figure 1:**
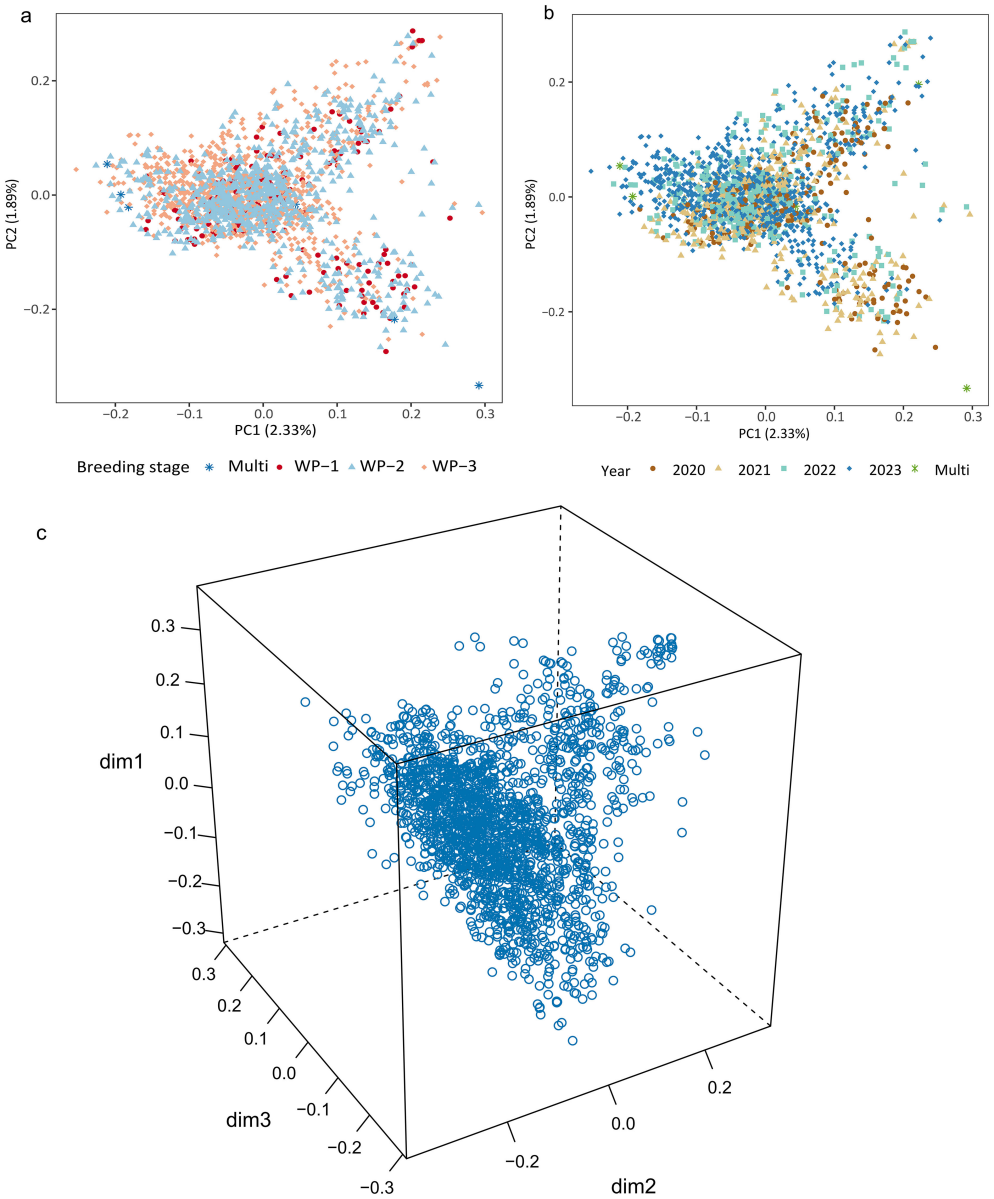
Illustrating the underlying population structure of the population under study. **(a, b)** illustrates principal coordinate analysis (PCoA) based on the Rogers’ distance matrix. The x- and y-axes represent the respective principal coordinates (PCs), with the values in parentheses indicating the percentage of variance explained by each PC. In **(a)** colors and shapes indicate the breeding stage of origin, with “Multi” in the legend denoting the common check genotypes. In **(b)** colors and shapes represent the year of origin, while “Multi” again denotes the check genotypes. **(c)** Three-dimensional multidimensional scaling (3D MDS) plot based on allele sharing distance (ASD), calculated using the “AWclust” R package, illustrating the genetic relationships among genotypes in the first three dimensions (Dim1–Dim3). Across all the panels each point represents one genotype.

The first and second principal coordinates (PC) explained 2.33% and 1.89% respectively, of the total variation. Plots of the PCs did not reveal clear clustering of genotypes according to breeding trial stage or year of origin (**Figures 1a, b**). This indicates an absence of an apparent population structure among the evaluated genotypes. Consistently, the three-dimensional multidimensional scaling (3D MDS) analysis based on allele sharing distance also showed no distinct grouping patterns (**Figure 1c**). In agreement with these results, the mean pairwise Rogers’ distance within each breeding stage and across all pairs of breeding stages ranged from 0.36 to 0.37 (**Supplementary Table 3**). Similarly, the mean pairwise Rogers’ distance within each year and across all pairs of years also ranged from 0.36 to 0.37 (**Supplementary Table 4**).

### 3.2 Analysis strategy 1: ignoring breeding stage effect leads to biased estimation of BLUEs

In the first stage of Strategy I, six environments were excluded due to repeatability values close to zero, including five for grain yield and one for plant height (**Supplementary Table 5**). The remaining environments showed high repeatability, with values ranging from 0.71 to 0.86 for grain yield, 0.77 to 0.94 for plant height, and 0.86 to 0.91 for heading date (**Supplementary Figure 2**).

**Figure 2:**
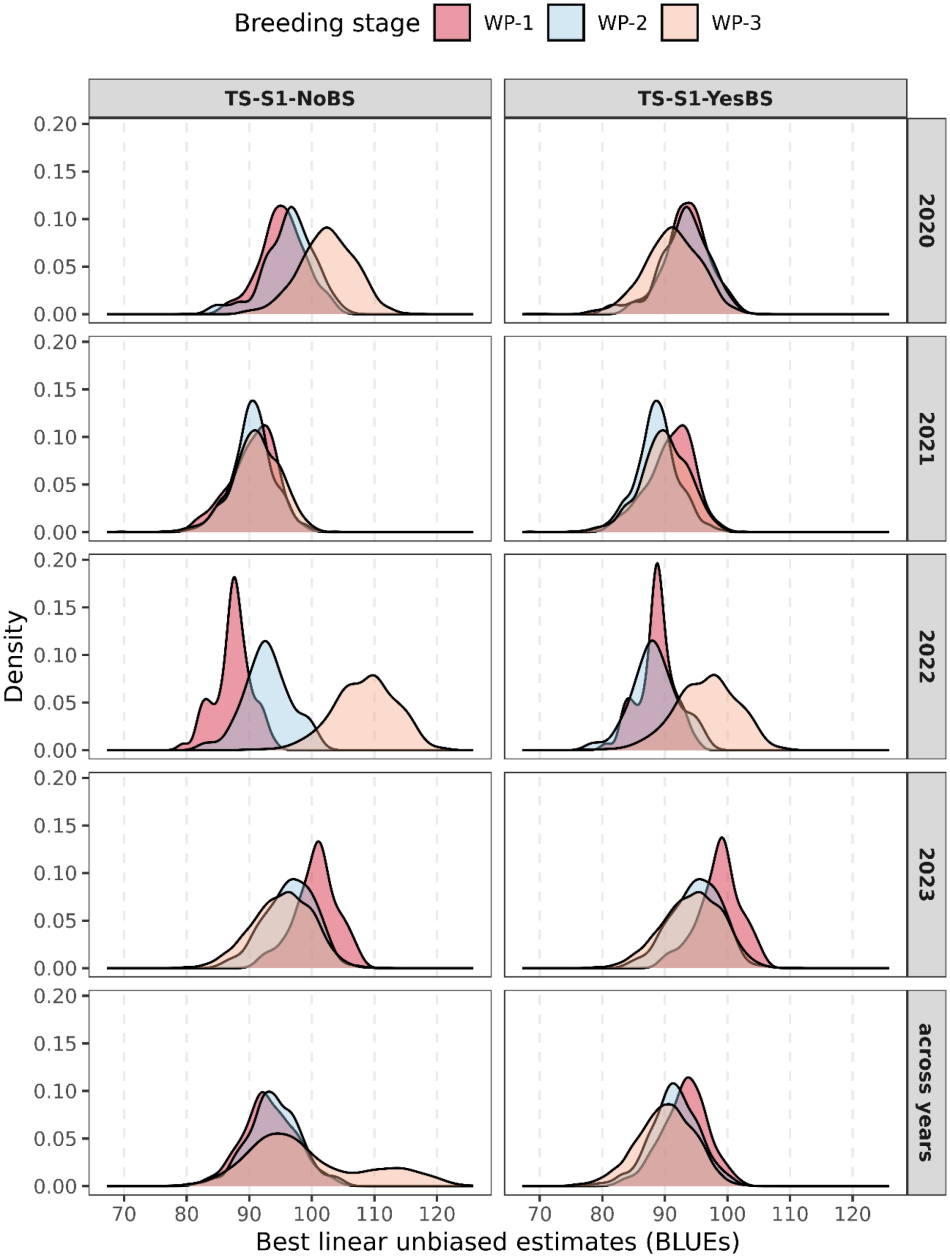
Distribution of Best Linear Unbiased Estimations (BLUEs) obtained from the second stage of analysis strategy 1 using models that either included the breeding stage effect in the environmental term (TS-S1-YesBS) or excluded it (TS-S1-NoBS) across years for the trait grain yield. Colors indicate the breeding stage from which the genotypes originate.

In the second stage, across traits and years, higher genotypic and error variances, as well as lower environmental variance, were observed when using the standard model (TS-S1-NoBS), in which environment was defined as year × location combination, compared to the non-standard model (TS-S1-YesBS), in which environment was defined as year × location × breeding stage combination (**Table 1**). We further observed that when the breeding stage is not included, its variance is partly assigned to the genotypic and error variances (**Table 1**). For grain yield, especially in 2022, the variance components from TS-S1-NoBS were unusually high compared to other years. This affected the across-year estimates (**Table 1**). For plant height, a clear drop in genotypic variance was observed under TS-S1-YesBS in 2022 and 2023, as reflected in the across-year results (**Table 1**). In contrast, for heading date, variance partitioning was largely similar between the two models. However, variance components could not be estimated for heading date in 2022 under TS-S1-YesBS because only two breeding stages (WP-1 and WP-3) were present, and there were no overlapping genotypes (**Table 1**). Consequently, the BLUEs from TS-S1-NoBS were biased, as evidenced by their clear stratification by breeding stage in 2022 for all traits (**Figure 2**; **Supplementary Figures 3**, **4**). In contrast, TS-S1-YesBS provided more reliable BLUEs. However, due to the lack of overlapping genotypes between WP-3 and the other breeding stages some stratification remained in 2022, albeit less pronounced. Heritability estimates were generally similar within and across years, except when the breeding stage effect was strong. This led to lower estimates under TS-S1-YesBS than under TS-S1-NoBS (**Supplementary Figure 5**). Overall, a clear breeding stage effect was observed. If not properly accounted for, this effect can lead to biased BLUEs.

**Table 1:** The genotypic variance ( $\sigma_{g}^{2}$), environment variance ( $\sigma_{env}^{2}$), and residual variance ( $\sigma_{\epsilon }^{2}$) estimated from models with the environmental effect either including the breeding stage (TS-S1-YesBS) or excluding it (TS-S1-NoBS) for grain yield, heading date, and plant height, across years from analysis strategy 1.

Trait	$\sigma_{g}^{2}$	$\sigma_{env}^{2}$	$\sigma_{\epsilon }^{2}$	Model	Year
Grain yield	21.73	291.41	36.96	TS-S1-NoBS	Across years
Grain yield	12.37	304.16	24.85	TS-S1-YesBS	Across years
Grain yield	16.78	244.19	35.23	TS-S1-NoBS	2020
Grain yield	13.61	296.96	23.21	TS-S1-YesBS	2020
Grain yield	10.40	320.50	15.36	TS-S1-NoBS	2021
Grain yield	10.52	264.53	14.89	TS-S1-YesBS	2021
Grain yield	53.96	302.04	57.48	TS-S1-NoBS	2022
Grain yield	13.95	375.45	30.30	TS-S1-YesBS	2022
Grain yield	11.01	308.29	32.71	TS-S1-NoBS	2023
Grain yield	10.35	282.44	32.02	TS-S1-YesBS	2023
Heading date	3.86	29.23	1.14	TS-S1-NoBS	Across years
Heading date	3.73	24.86	1.12	TS-S1-YesBS	Across years
Heading date	2.97	2.20	0.60	TS-S1-NoBS	2021
Heading date	2.97	2.20	0.60	TS-S1-YesBS	2021
Heading date	3.31	19.49	0.76	TS-S1-NoBS	2022
Heading date	5.12	7.28	1.41	TS-S1-NoBS	2023
Heading date	4.79	5.03	1.37	TS-S1-YesBS	2023
Plant height	20.83	99.24	14.20	TS-S1-NoBS	Across years
Plant height	16.06	108.25	13.03	TS-S1-YesBS	Across years
Plant height	18.49	26.00	8.95	TS-S1-NoBS	2021
Plant height	18.44	26.11	8.95	TS-S1-YesBS	2021
Plant height	19.77	124.82	19.36	TS-S1-NoBS	2022
Plant height	11.64	105.61	17.31	TS-S1-YesBS	2022
Plant height	23.24	169.04	14.98	TS-S1-NoBS	2023
Plant height	16.65	152.05	13.72	TS-S1-YesBS	2023

### 3.3 Analysis strategy 2: differential performance of check genotypes across breeding stages

In Strategy 2, stage one, the heritability estimates for grain yield ranged from 0.76 to 0.89 in WP-1, 0.76 to 0.86 in WP-2, and 0.57 to 0.83 in WP-3. For plant height, heritability ranged from 0.89 to 0.96 in WP-1 and from 0.76 to 0.97 in WP-2, while in WP-3 it was 0.84. For heading date, heritability ranged from 0.88 to 0.96 in WP-1 and from 0.92 to 0.94 in WP-3 (**Table 2**).

**Table 2:** Results from stage one of analysis strategy 2 showing the genotypic variance ( $\sigma_{g}^{2}$), genotype by location variance ( $\sigma_{gl}^{2}$), and residual variance ( $\sigma_{\epsilon }^{2}$), along with the average number of locations per genotype (N_loc_), the average number of replications per genotype (N_rep_), and heritability estimates for grain yield, heading date, and plant height across years and breeding stages. NA in the table means not applicable.

Trait	Year	Breeding stage	$\sigma_{g}^{2}$	$\sigma_{gl}^{2}$	$\sigma_{\epsilon }^{2}$	N_loc_	N_rep_	Heritability
Grain yield	2020	WP-1	11.03	15.98	13.35	15.42	1.96	0.88
Grain yield	2021	WP-1	8.76	38.42	8.95	15.44	2.00	0.76
Grain yield	2022	WP-1	7.40	9.14	8.78	15.47	1.99	0.89
Grain yield	2023	WP-1	6.68	11.40	13.56	13.46	1.95	0.83
Grain yield	2020	WP-2	9.44	8.76	12.68	6.00	1.99	0.79
Grain yield	2021	WP-2	9.71	6.84	5.34	6.00	1.99	0.86
Grain yield	2022	WP-2	7.59	7.81	6.07	5.99	1.71	0.80
Grain yield	2023	WP-2	8.71	7.87	11.93	4.99	1.97	0.76
Grain yield	2020	WP-3	14.55	NA	22.87	NA	5.61	0.78
Grain yield	2021	WP-3	12.37	NA	14.37	NA	3.34	0.74
Grain yield	2022	WP-3	16.94	NA	15.48	NA	4.34	0.83
Grain yield	2023	WP-3	11.08	NA	27.58	NA	3.33	0.57
Heading date	2021	WP-1	1.70	0.40	0.41	4.55	1.98	0.93
Heading date	2022	WP-1	1.86	0.81	0.40	4.11	1.98	0.88
Heading date	2023	WP-1	3.47	0.57	0.59	6.63	1.37	0.96
Heading date	2022	WP-3	3.38	NA	0.48	NA	2.17	0.94
Heading date	2023	WP-3	4.33	NA	1.19	NA	3.34	0.92
Plant height	2021	WP-1	28.62	4.39	5.59	5.65	1.80	0.96
Plant height	2022	WP-1	12.29	6.50	8.39	7.23	1.98	0.89
Plant height	2023	WP-1	16.84	8.41	14.20	7.65	1.76	0.89
Plant height	2021	WP-2	21.56	NA	1.28	NA	2.24	0.97
Plant height	2022	WP-2	15.07	11.98	4.94	3.00	1.99	0.76
Plant height	2023	WP-2	18.91	1.71	11.40	2.99	1.50	0.86
Plant height	2021	WP-3	20.69	NA	8.50	NA	2.23	0.84
Plant height	2023	WP-3	17.49	NA	7.65	NA	2.23	0.84

**Figure 3:**
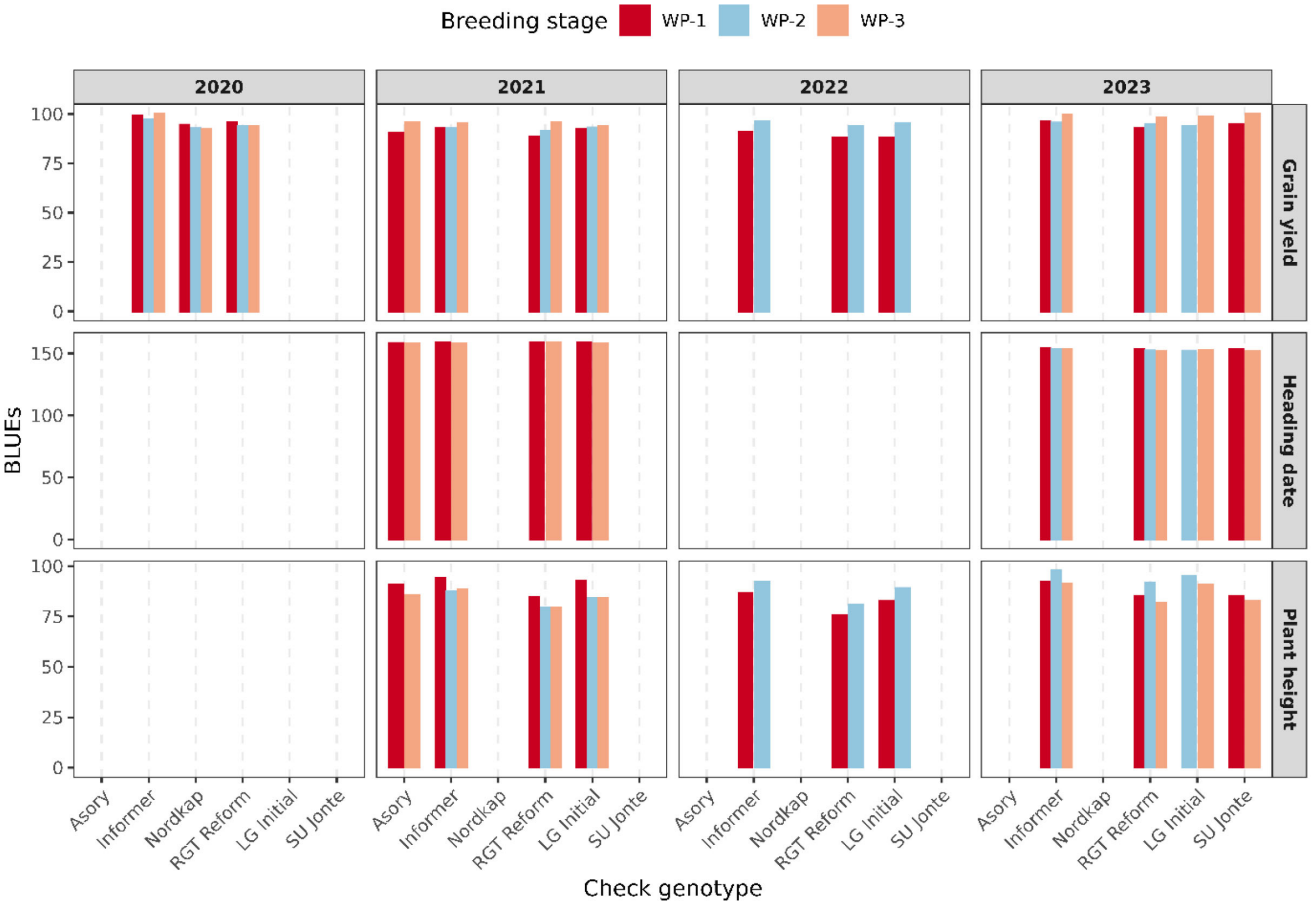
From the stage one of analysis strategy 2, the trait performance (BLUEs, y-axis) of check genotypes (x-axis) across breeding stages (indicated by colors in the legend), across traits (horizontal facets) and years (vertical facets).

The BLUEs of the three to four common check genotypes across breeding stages within each year revealed consistent differences in performance depending on the breeding stage, except for heading date and grain yield in 2020 (**Figure 3**). Systematic differences in grain yield were observed for checks in WP-3 in 2022 and 2023, and in WP-2 in 2022. Such differences were also observed for plant height in WP-1 in 2021 and in WP-2 in 2022 and 2023. Accordingly, BLUEs estimated from models that included breeding stage (TS-S2-YesBS) differed from those estimated from models that did not (TS-S2-NoBS), resulting in different degrees of clustering of BLUEs by breeding stage (**Supplementary Figures 6**–**8**).

The genotype × location interaction variance for grain yield in WP-1 was more than 1.5 times higher than the genotypic variance, while in WP-2 it was close to the genotypic variance (**Table 2**). This is expected since WP-1 includes a larger number of locations than WP-2. In contrast, for traits such as plant height and heading date, the genotype × location variance was lower than the genotypic variance, reflecting their simpler genetic control and lower environmental sensitivity (**Table 2**).

### 3.4 Choice of the second-stage phenotypic analysis model does not impact prediction ability in 5-fold CV

**Figure 4:**
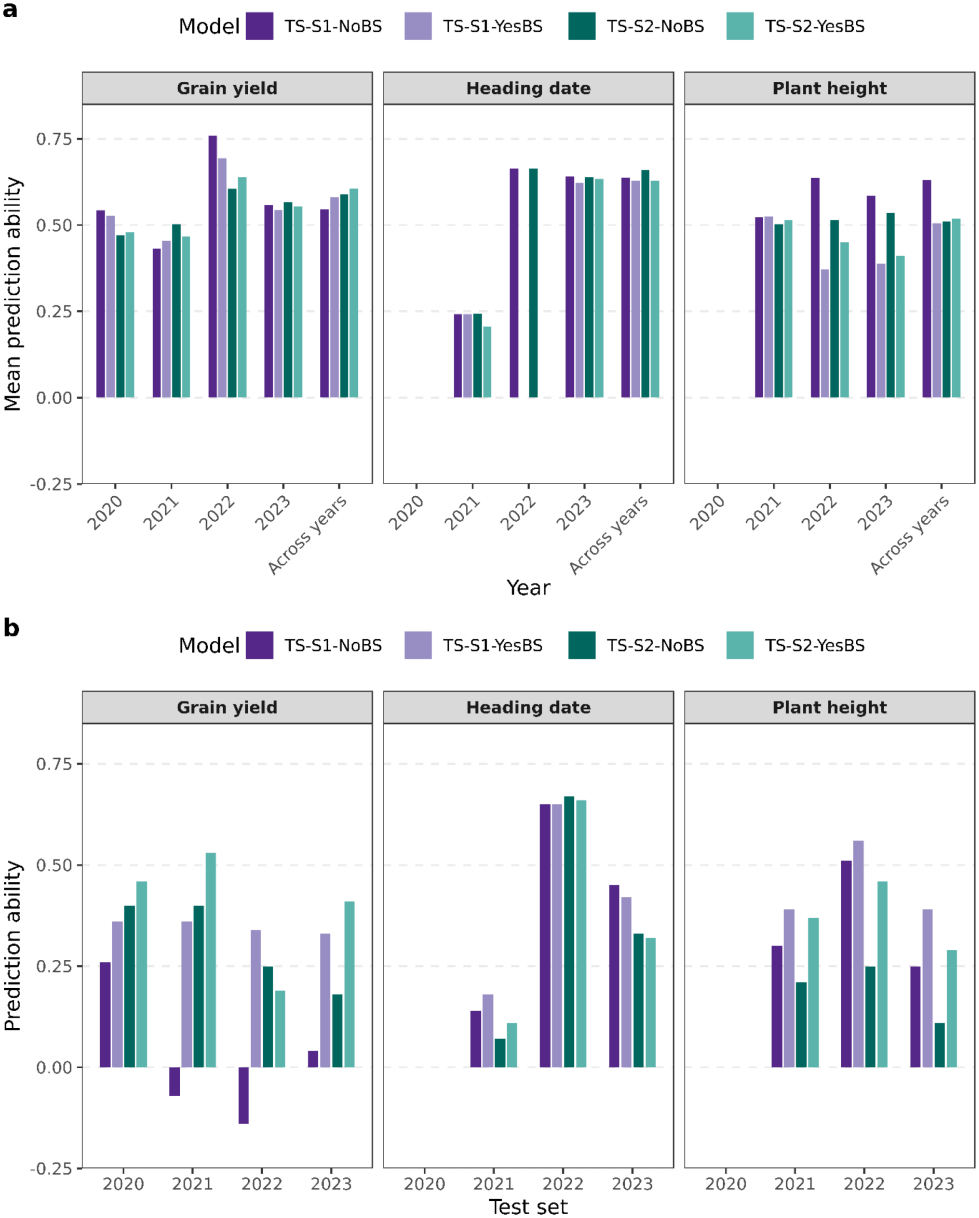
Illustration of **(a)** 5-fold cross validations (5-fold CV) and **(b)** leave one year out cross validation (LOY-CV) results across the analysis strategies and traits. **(a)** Mean prediction ability (y-axis) using within each year and across years BLUEs (x-axis) from 5-fold CV for grain yield, heading date, and plant height comparing models with (TS-S1-YesBS, TS-S2-YesBS) and without (TS-S1-NoBS, TS-S2-NoBS) breeding stage in the model represented by colors in the legend. **(b)** Prediction ability (y-axis) across different test set years (Test set, x-axis) from LOY-CV for grain yield, heading date, and plant height, comparing models with (TS-S1-YesBS, TS-S2-YesBS) and without (TS-S1-NoBS, TS-S2-NoBS) breeding stage in the model represented by colors in the legend.

The four models used in the second stage across the two analysis strategies exhibited varying prediction abilities depending on the trait in the 5-fold CV (**Figure 4a**; **Supplementary Tables 6**, **7**). Within each year 5-fold CV revealed that, for grain yield, there is no model that is consistently performing better than the other models (**Figure 4a**). However, for the plant height, the standard second stage model (TS-S1-NoBS) consistently showed higher prediction ability than the other models (**Figure 4a**). But, for heading date, all models performed almost similarly (**Figure 4a**). 5-fold CV on across years BLUEs revealed that, all non-standard models showed higher prediction ability than TS-S1-NoBS for grain yield, whereas the opposite trend was observed for plant height. For heading date, the models showed almost similar performance (**Figure 4a**). Overall, the mean prediction abilities were moderate to high for all traits regardless of the model used, in both within and across years 5-fold CV (**Supplementary Tables 6**, **7**). For grain yield, the mean prediction ability exceeded 0.43 within years 5-fold CV and 0.55 across years 5-fold CV. For heading date, the mean prediction ability was mostly above 0.60 except in 2021, in both the within and across years 5-fold CV. For plant height, the mean prediction ability was above 0.37 within years 5-fold CV and 0.51 across years 5-fold CV (**Supplementary Tables 6**, **7**). In summary, across all the models no abnormally high or low prediction abilities were observed.

### 3.5 TS-S1-YesBS is the most reliable second-stage model for predicting genotype performance in a year

**Figure 5:**
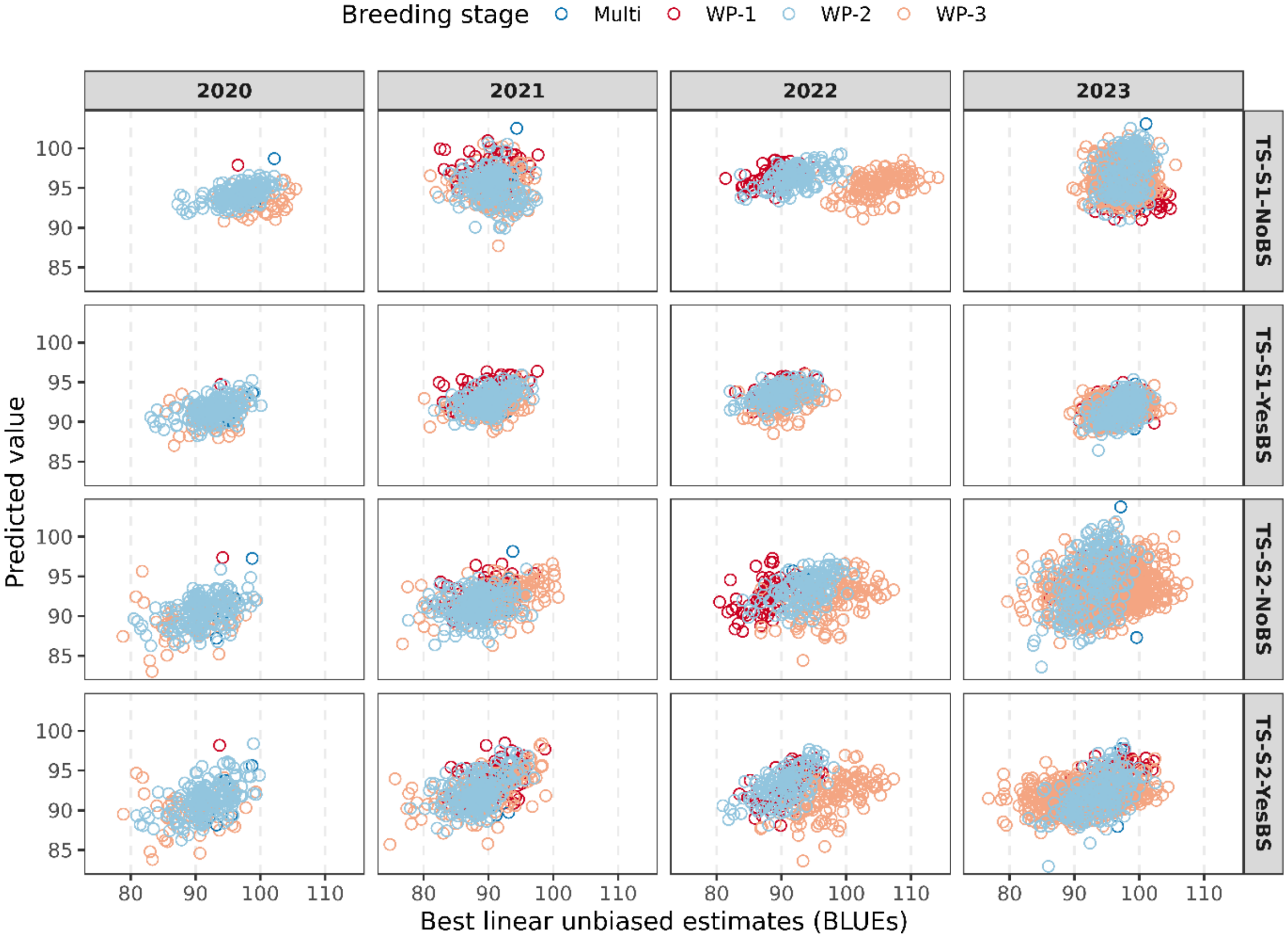
Scatter plots of observed BLUEs versus predicted values for grain yield across test years, comparing models that include breeding stage effects (–YesBS) and those that exclude them (–NoBS) under analysis strategy 1 (TS-S1) and analysis strategy 2 (TS-S2). Points are colored by breeding stage, with “Multi” indicating genotypes present in multiple breeding stages (i.e., check genotypes).

In the LOY-CV, the choice of the second-stage analysis model strongly affected prediction ability for grain yield. The standard TS-S1-NoBS model, which excludes the breeding stage in defining the environment term, resulted in very low to negative prediction abilities for most years, except 2020 (2021: −0.14, 2022: −0.07, 2023: 0.04) (**Figure 4b**, **Supplementary Table 8**). In contrast, when breeding stage was included in the environment term (TS-S1-YesBS), these abnormal prediction abilities were not observed (**Figure 4b**, **Supplementary Table 8**). When the breeding stage was used as the analysis unit (Strategy 2), no such low prediction abilities were observed, regardless of whether breeding stage was included in the second-stage model (**Figure 4b**, **Supplementary Table 9**). Similar to Strategy 1, the model that accounted for the breeding stage (TS-S2-YesBS) had a higher prediction ability than TS-S2-NoBS model. Overall, the TS-S2-YesBS model resulted in the highest prediction ability for grain yield. No negative prediction abilities were observed for plant height and heading date across the models (**Figure 4b**; **Supplementary Tables 8**, **9**). For plant height, models that included breeding stage consistently showed higher prediction ability than those that did not across both strategies, with TS-S1-YesBS resulting the highest prediction ability. In contrast, for heading date, models from Strategy 1 (TS-S1-NoBS and TS-S1-YesBS) showed better prediction ability than those from Strategy 2 (**Figure 4b**; **Supplementary Tables 8**, **9**). However, scatter plots of BLUEs and predicted values showed clear grouping by breeding stage in all models across the traits except TS-S1-YesBS, especially in years where the breeding stage effect was strong, such as 2022 (**Figure 5**; **Supplementary Figures 9**, **10**). In summary, TS-S1-YesBS produces more accurate BLUEs and stable prediction ability across traits.

### 3.6 Prediction ability of the standard model is biased under LOBS-CV

**Figure 6:**
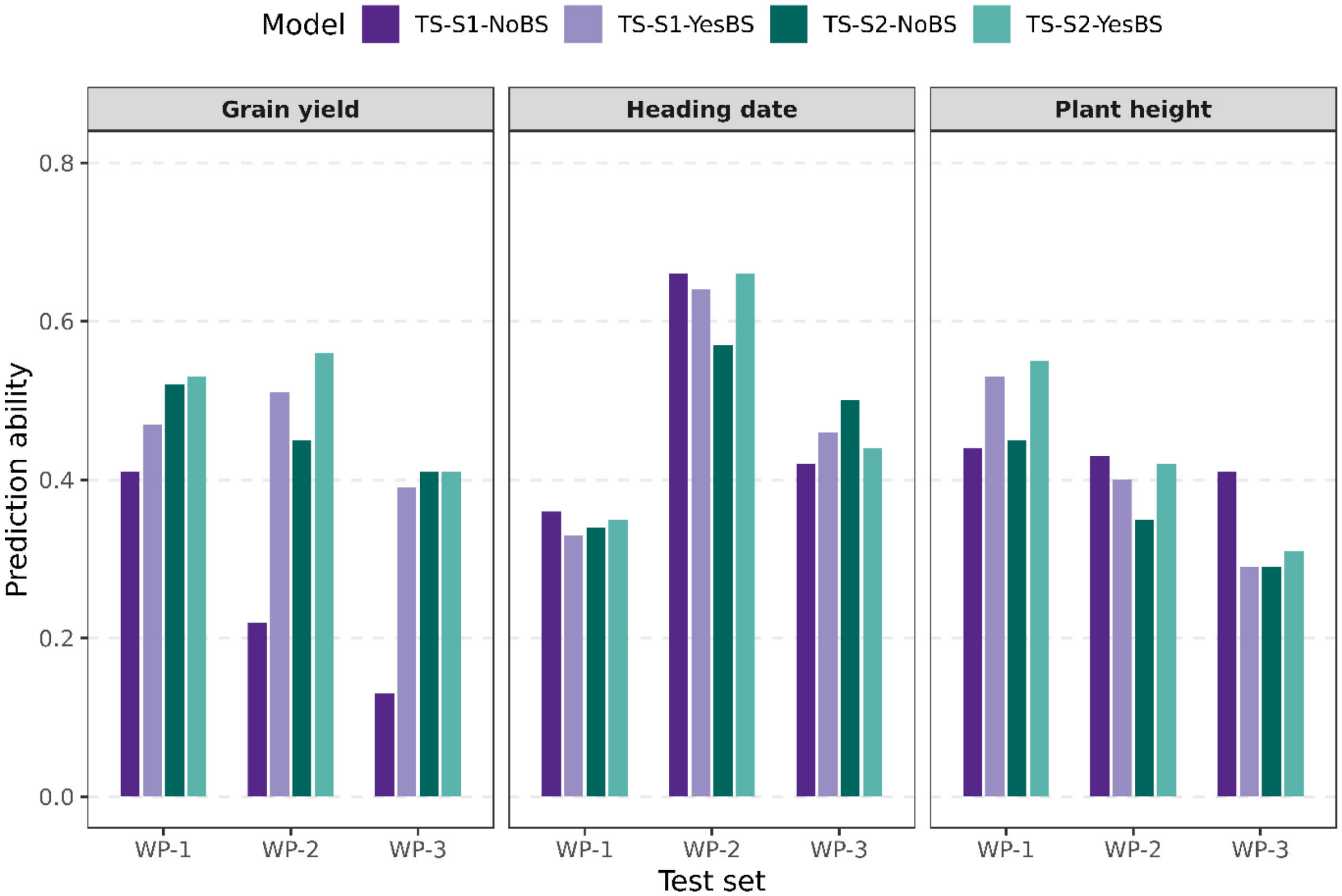
Prediction ability (y-axis) across different test set breeding stages (Test set, x-axis) from leave-one-breeding stage-out cross-validation for grain yield, heading date, and plant height comparing models with (TS-S1-YesBS, TS-S2-YesBS) and without (TS-S1-NoBS, TS-S2-NoBS) breeding stage in the model represented by colors in the legend.

In the LOBS-CV, for grain yield, the standard model (TS-S1-NoBS) resulted in abnormally low prediction abilities when predicting WP-2 and WP-3 using the remaining breeding stages (**Figure 6**). In contrast, for the other traits, no abnormal prediction abilities were observed, regardless of the model used (**Figure 6**). However, scatter plots of BLUEs and predicted values showed clear grouping when predicting the WP-2 and WP-3 using the rest of the breeding stages using the standard model for the grain yield and plant height (**Supplementary Figures 11**–**13**). The clusters in the WP-2 are due to biased predicted values and where as in the WP-3 it is due to the biased BLUEs for both the grain yield and plant height.

## 4 Discussion

In breeding datasets, it is common to have multiple breeding stages evaluated within the same environment, as breeding programs often assess trials from preliminary to advanced stages originating from different breeding cycles together. In addition, breeding companies may use different check genotypes across breeding cycles, which can lead to poor connectivity among breeding stages within a year and also across years (Bernal-Vasquez et al., 2014). Beyond this, even when trials are conducted in the same location and year, the actual growing conditions across breeding stages may not be identical. Differences in soil fertility, field position, pre-crop history, or crop management can introduce systematic environmental differences among stages (Sosibo et al., 2017; Wang et al., 2018; Chandio et al., 2023; Groeneveld et al., 2024). Consequently, some breeding stages may be grown under more favourable conditions than others, leading to consistent differences in genotype performance across stages within the same environment (**Supplementary Figures 14**, **15**). Our phenotypic data analysis results across the two strategies clearly support this, as systematic stratification of BLUEs by breeding stage was observed, particularly for grain yield and plant height in years with a strong breeding stage effect. Because these breeding stages originate from different breeding cycles, such systematic stratification is unlikely to be driven by directional selection. Rather, it is more plausibly explained by the environmental differences to which the different breeding stages were subjected.

Findings from analysis strategy 1 indicate that treating all breeding stages within a year × location combination as a single environment in the standard two-stage analysis (TS-S1-NoBS) leads to biased BLUEs when breeding stage effects are ignored. In contrast, accounting for breeding stage in the second-stage model (TS-S1-YesBS) produced more reliable across-environment BLUEs for most years. The only notable exception was WP-3 in 2022, where no overlapping genotypes were available with the other breeding stages within that year, limiting the model’s ability to fully correct for the breeding stage effect. However, this issue was largely resolved in the across-year analysis because WP-3 in 2022 shared at least one overlapping genotype with WP-3 in other years, providing the necessary connection to adjust for stage effects and resulting in more accurate BLUEs across years (**Figure 2**; **Supplementary Figures 3**, **4**). This highlights the importance of general principle of connectivity in non-orthogonal data for accurate BLUEs calculation, and supports the use of consistent check genotypes used across breeding cycles (Piepho and Laidig, 2025).

Furthermore, previous studies have shown that the way phenotypic data are analysed and model effects are fitted can influence the accuracy of BLUEs (Bernal-Vasquez et al., 2014). Therefore, we used breeding stage as analysis unit in the strategy 2. When shifting the analysis unit from environment to breeding stage within a year (Strategy 2), the clustering of BLUEs by breeding stage was reduced, although not completely removed, when breeding stage was not included in the model (**Supplementary Figures 6**–**8**). This is because breeding stages are evaluated across multiple locations, and not all locations are equally affected by unfavourable conditions. The presence of overlapping genotypes across locations allows correction of location-specific effects, which is not possible when each environment is analysed separately. This observation is consistent with previous studies demonstrating that multi-location testing with sufficient genotype overlap improves the estimation of environmental effects and accounts for site heterogeneity (Cullis et al., 2000; Ortiz et al., 2023). However, some variation due to breeding stage still remains, and ignoring it continues to affect the accuracy of BLUEs.

Overall, both analysis strategies clearly demonstrate the presence of confounding environmental effects associated with breeding stage in the estimated BLUEs. Accounting for this effect is therefore essential to obtain accurate BLUEs, and ignoring it can negatively affect breeding progress when phenotype-based selection is applied. Moreover, it is noteworthy that, irrespective of the models used and the presence of environmental effects associated with breeding stage, medium to high repeatability and heritability values were observed across grain yield, plant height, and heading date, (**Supplementary Figures 2**, **5**; **Table 2**) falling within the range of values previously reported for wheat (Isidro et al., 2015; Gudeta et al., 2025; Lell et al., 2025; Mullualem et al., 2025). Phenotypic data-based heritability values are often interpreted as indicators of phenotypic data quality (Schmidt et al., 2019). However, our results highlight that heritability values alone do not provide a complete picture of the quality and reliability of estimated BLUEs. Therefore, while heritability can serve as an important measure for assessing phenotypic data quality, it should not be considered the sole criterion for evaluating the reliability of estimated BLUEs.

Even though no abnormally low or high mean prediction abilities were observed in the 5-fold CV using BLUEs from any of the four second-stage models, a clear pattern was evident. For grain yield (in 2020 and 2022) and plant height (in 2022 and 2023), which are years with a strong breeding stage effect, the mean prediction ability of the standard second-stage model (TS-S1-NoBS) was overestimated (**Figure 4**). This overestimation is likely driven by clustering of observations by breeding stage (**Supplementary Figure 16**). Because 5-fold CV randomly splits the data, each fold contains samples from all breeding stages (Ahmed, 2024), and both training and test sets share the same stage-specific structure (**Supplementary Tables 10**, **11**). As a result, predictions capture this clustering rather than true genotypic differences, leading to inflated correlations between observed and predicted values (**Supplementary Figure 16**) (Werner et al., 2020). Furthermore, unlike the mean prediction ability, the standard errors remained almost similar across the different models, even in years with a strong breeding-stage effect, because the same systematic stage-specific structure was retained in every fold of the 5-fold CV (**Supplementary Tables 6**, **7**). Therefore, caution is needed when interpreting mean prediction abilities in the presence of stratification in the BLUEs, as it may give a false impression of strong agreement between phenotypic and genotypic data, while the effect is mainly driven by breeding stage clustering.

The impact of breeding stage is further evident when predicting genotype performance within a year using the data from the other years. Grain yield, which showed a strong breeding stage effect, was also the most affected trait when the standard two-stage analysis was used. In general, across the traits, when breeding stage was not included in the model, clustering effects in either the training or test set led to biased prediction ability, depending on whether years with strong breeding stage effects were present in the training or test data (**Figure 5**; **Supplementary Figures 9**, **10**). Similar clustering-driven bias was also observed when predicting one breeding stage using the remaining stages (**Supplementary Figures 11**-**13**). These results show that using biased BLUEs in genomic prediction can further propagate errors and lead to unreliable predictions. Furthermore, the absence of a clear population structure based on either year of origin or breeding stage of origin (**Figure 1**) further strengthens our interpretation that the unreliable predictions observed in this study can be attributed to inaccurate BLUEs rather than to underlying population structure.

Therefore, correcting for breeding stage effects is essential to obtain accurate BLUEs for both phenotype-based selection and genomic prediction. More importantly, our results support the basic principle that prediction ability alone should not be used to select a phenotypic analysis strategy. As higher prediction ability does not necessarily indicate more accurate phenotypic estimates, as it may be artificially inflated by residual clustering or confounding effects in the phenotypic data (Bernal-Vasquez et al., 2014). Therefore, prediction ability should always be interpreted together with diagnostic plots comparing observed (BLUEs) and predicted values to assess the presence of clustering or other systematic biases before drawing conclusions about model performance (Bernal-Vasquez et al., 2014).

Based on these findings, we recommend using sufficient check genotypes within and across breeding cycles to improve connectivity and allow proper correction of breeding stage and year effects. Among the evaluated approaches, the modified standard two-stage model that includes breeding stage is the most reliable for accurate BLUE estimation and prediction. In addition, using breeding stage within a year as the unit of analysis (Strategy 2) may be beneficial for complex traits such as grain yield, only when breeding cycles are well connected, as prediction abilities were generally higher under Strategy 2 than Strategy 1 in LOY-CV and LOBS-CV when breeding stage was included (**Figures 4b**, **6**; **Supplementary Tables 8**, **9**). A likely reason is that Strategy 2 allows explicit modelling of genotype × environment interaction within replicated breeding stages (WP-1 and WP-2), and separating this interaction from the genotypic effect can improve prediction ability for complex traits such as grain yield (Dawson et al., 2013; Jarquín et al., 2014). If breeding cycles are poorly connected, using breeding stage as the analysis unit may still be insufficient to fully account for breeding stage effects. Therefore, we do not recommend this approach as a universal analysis strategy, as its effectiveness depends on the degree of connectivity and trait complexity among breeding cycles (**Figure 5**; **Supplementary Figures 9**, **10**).

## 5 Conclusion

Accurate estimation of BLUEs is essential for both phenotypic selection and genomic prediction to achieve sustained genetic gain in breeding programs. In genomic prediction, the quality of predicted values depends strongly on the size of the training set and the quality of the phenotypic data. Therefore, combining phenotypic data across breeding cycles and years is important to build a robust training set. However, when systematic breeding stage effects arise due to differences in growing conditions, relying on the standard two-stage analysis can lead to biased estimates. Our results show that explicitly accounting for breeding stage in the model is necessary to obtain accurate BLUEs, which in turn supports reliable genomic prediction and effective selection decisions.

## Data Availability

The datasets and results generated during the current study are presented in the figures, tables, and supplementary materials of this manuscript. The curated genotypic and phenotypic datasets analyzed during the study are available for corresponding author upon reasonable request.

## Supplementary material

Supplementary Figure 1 Schematic representation of the cross-validation strategies: **(a)** 5-fold cross-validation and **(b)** leave-one-year-out cross-validation.

Supplementary Figure 2 Distribution of repeatability estimates (y-axis) across traits and years (x-axis) from stage one of analysis strategy 1. The red dashed line indicates the repeatability threshold of 0.3.

Supplementary Figure 3 Distribution of BLUEs obtained from stage two of analysis strategy 1 using models that either included the breeding stage effect in the environmental term (TS-S1-YesBS) or excluded it (TS-S1-NoBS) across years for the trait plant height. Colors indicate the breeding stage from which the genotypes originate.

Supplementary Figure 4 Distribution of BLUEs obtained from stage two of analysis strategy 1 using models that either included the breeding trial stage effect in the environmental term (TS-S1-YesBS) or excluded it (TS-S1-NoBS) across years for the trait heading date. Colors indicate the breeding stage from which the genotypes originate.

Supplementary Figure 5 Heritability estimates (x-axis) from analysis Strategy 1 across traits and years (y-axis). Colored bars indicate models with (TS-S1-YesBS) and without (TS-S1-NoBS) breeding stage in the environmental effect.

Supplementary Figure 6 Distribution of BLUEs obtained from stage two of analysis strategy 2 using models that either included the breeding trial stage effect in the model (TS-S2-YesBS) or excluded it (TS-S2-NoBS) across years for the trait grain yield. Colors indicate the breeding stage from which the genotypes originate.

Supplementary Figure 7 Distribution of BLUEs obtained from stage two of analysis strategy 2 using models that either included the breeding trial stage effect in the model (TS-S2-YesBS) or excluded it (TS-S2-NoBS) across years for the trait plant height. Colors indicate the breeding stage from which the genotypes originate.

Supplementary Figure 8 Distribution of BLUEs obtained from stage two of analysis strategy 2 using models that either included the breeding trial stage effect in the model (TS-S2-YesBS) or excluded it (TS-S2-NoBS) across years for the trait heading date. Colors indicate the breeding stage from which the genotypes originate.

Supplementary Figure 9 Scatter plots of observed BLUEs versus predicted values for plant height across test years, comparing models that include breeding stage effects (–YesBS) and those that exclude them (–NoBS) under analysis strategy 1 (TS-S1) and analysis strategy 2 (TS-S2). Points are colored by breeding stage, with “Multi” indicating genotypes present in multiple breeding stages (i.e., check genotypes).

Supplementary Figure 10 Scatter plots of observed BLUEs versus predicted values for heading date across test years, comparing models that include breeding stage effects (–YesBS) and those that exclude them (–NoBS) under analysis strategy 1 (TS-S1) and analysis strategy 2 (TS-S2). Points are colored by breeding stage, with “Multi” indicating genotypes present in multiple breeding stages (i.e., check genotypes).

Supplementary Figure 11 Scatter plots of observed BLUEs versus predicted values for grain yield across test breeding stages, comparing models that include breeding stage effects (–YesBS) and those that exclude them (–NoBS) under analysis strategy 1 (TS-S1) and analysis strategy 2 (TS-S2). The red colored circles indicates the clusters.

Supplementary Figure 12 Scatter plots of observed BLUEs versus predicted values for plant height across test breeding stages, comparing models that include breeding stage effects (–YesBS) and those that exclude them (–NoBS) under analysis strategy 1 (TS-S1) and analysis strategy 2 (TS-S2). The red colored circles indicates the clusters

Supplementary Figure 13 Scatter plots of observed BLUEs versus predicted values for heading date across test breeding stages, comparing models that include breeding stage effects (–YesBS) and those that exclude them (–NoBS) under analysis strategy 1 (TS-S1) and analysis strategy 2 (TS-S2).

Supplementary Figure 14 Distribution of within-environment BLUEs for environments with more than one breeding stage across years for grain yield: **(a)** 2020, **(b)** 2021, **(c)** 2022, and **(d)** 2023. Different colors indicate different breeding stages. The distributions show that, in some environments, there are clear systematic differences in performance among breeding stages.

Supplementary Figure 15 Distribution of within-environment BLUEs for environments with more than one breeding stage across years for plant height: **(a)** 2021, **(b)** 2022, and **(c)** 2023; and for heading date: **(d)** 2023. Different colors indicate different breeding stages. The distributions show that, in some environments, there are clear systematic differences in performance among breeding stages.

Supplementary Figure 16 Scatter plots showing BLUEs (x-axis) versus predicted values (y-axis) for grain yield in **(a)** 2021 (a year with no observed breeding-stage effect) and **(b)** 2022 (a year with a strong breeding-stage effect) using the TS-S1-NoBS model across the initial five runs of the 5-fold cross-validation. The r-values in each subplot represent the correlation between BLUEs and predicted values (prediction ability). The presence of clustering by breeding stage (indicated by coloured points) in **(b)** suggests that, in the presence of a breeding-stage effect, the TS-S1-NoBS model produces BLUEs that are confounded by environmental effects associated with breeding stage, leading to an overestimation of prediction ability in each run. This pattern is not observed when the breeding-stage effect is absent or negligible, as shown in **(a)**.

Supplementary Table 1 Summary of the number of genotypes with phenotypic observations (n genotypes), genotypes with both genotypic and phenotypic data (n genotypes GP), and the number of test locations (n locations), stratified by year, trait, and breeding stage.

Supplementary Table 2 Summary of the number of genotypes genotyped with the 7K and 25K marker chips across different years.

Supplementary Table 3 The mean and standard deviation (SD) of the Rogers’ distance between the genotypes within the breeding trial stage and between the breeding trial stages.

Supplementary Table 4 The mean and standard deviation (SD) of the Rogers’ distance between the genotypes within the year and between the years.

Supplementary Table 5 Overview of the environments removed during stage one of the analysis strategy 1 across traits and years due to repeatability lower than 0.30.

Supplementary Table 6 Mean prediction ability across years from 5-fold cross-validation (analysis strategy 1) for grain yield, plant height, and heading date, comparing models with (TS-S1-YesBS) and without (TS-S1-NoBS) breeding stage in the environment effect.

Supplementary Table 7 Mean prediction ability across years from 5-fold cross-validation (analysis strategy 2) for grain yield, plant height, and heading date, comparing models with (TS-S2-YesBS) and without (TS-S2-NoBS) breeding stage effect.

Supplementary Table 8 Prediction ability across years (Test set) from leave-one-year-out cross-validation (analysis strategy 1) for grain yield, plant height, and heading date, comparing models with (TS-S1-YesBS) and without (TS-S1-NoBS) breeding stage in the environment effect.

Supplementary Table 9 Prediction ability across years (Test set) from leave-one-year-out cross-validation (analysis strategy 2) for grain yield, plant height, and heading date, comparing models with (TS-S2-YesBS) and without (TS-S2-NoBS) breeding stage effect.

Supplementary Table 10 Example table showing the proportion of genotypes from each breeding stage relative to the total number of genotypes in 2022. N represents the number of genotypes belonging to each breeding stage.

Supplementary Table 11 Example table showing the mean proportion of genotypes from each breeding stage in each fold across 50 runs of 5-fold cross-validation, relative to the total number of genotypes in 2022. This table shows that, in each fold, the average proportion of genotypes from each breeding stage is similar to the proportions observed in the base population (**Supplementary Table 10**). Therefore, if systematic clustering by breeding stage is present in the base population, it will also be reflected in each training and test set in every run of the 5-fold cross-validation, potentially leading to unreliable estimates of prediction ability.
