# Supplementary material for: Optimizing phenotypic data analysis strategies to enhance genomic prediction in winter wheat breeding
Source: Front Plant Sci. 2026 Aug 4;17:1898203. doi: 10.3389/fpls.2026.1898203 (PMC13481875; doi:10.3389/fpls.2026.1898203)
Supplement: Supplementary file 1 [file SupplementaryFile1.docx]

# **Supplementary figures**

Supplementary Figure 1: Schematic representation of the cross-validation strategies: (a) 5-fold cross-validation and (b) leave-one-year-out cross-validation.

Supplementary Figure 2: Distribution of repeatability estimates (y-axis) across traits and years (x-axis) from stage one of analysis strategy 1. The red dashed line indicates the repeatability threshold of 0.3.

Supplementary Figure 3: Distribution of BLUEs obtained from stage two of analysis strategy 1 using models that either included the breeding stage effect in the environmental term (TS-S1-YesBS) or excluded it (TS-S1-NoBS) across years for the trait plant height. Colors indicate the breeding stage from which the genotypes originate.

Supplementary Figure 4: Distribution of BLUEs obtained from stage two of analysis strategy 1 using models that either included the breeding trial stage effect in the environmental term (TS-S1-YesBS) or excluded it (TS-S1-NoBS) across years for the trait heading date. Colors indicate the breeding stage from which the genotypes originate.

Supplementary Figure 5: Heritability estimates (x-axis) from analysis Strategy 1 across traits and years (y-axis). Colored bars indicate models with (TS-S1-YesBS) and without (TS-S1-NoBS) breeding stage in the environmental effect.

Supplementary Figure 6: Distribution of BLUEs obtained from stage two of analysis strategy 2 using models that either included the breeding trial stage effect in the model (TS-S2-YesBS) or excluded it (TS-S2-NoBS) across years for the trait grain yield. Colors indicate the breeding stage from which the genotypes originate.

Supplementary Figure 7: Distribution of BLUEs obtained from stage two of analysis strategy 2 using models that either included the breeding trial stage effect in the model (TS-S2-YesBS) or excluded it (TS-S2-NoBS) across years for the trait plant height. Colors indicate the breeding stage from which the genotypes originate.

Supplementary Figure 8: Distribution of BLUEs obtained from stage two of analysis strategy 2 using models that either included the breeding trial stage effect in the model (TS-S2-YesBS) or excluded it (TS-S2-NoBS) across years for the trait heading date. Colors indicate the breeding stage from which the genotypes originate.

Supplementary Figure 9: Scatter plots of observed BLUEs versus predicted values for plant height across test years, comparing models that include breeding stage effects (–YesBS) and those that exclude them (–NoBS) under analysis strategy 1 (TS-S1) and analysis strategy 2 (TS-S2). Points are colored by breeding stage, with “Multi” indicating genotypes present in multiple breeding stages (i.e., check genotypes).

Supplementary Figure 10: Scatter plots of observed BLUEs versus predicted values for heading date across test years, comparing models that include breeding stage effects (–YesBS) and those that exclude them (–NoBS) under analysis strategy 1 (TS-S1) and analysis strategy 2 (TS-S2). Points are colored by breeding stage, with “Multi” indicating genotypes present in multiple breeding stages (i.e., check genotypes).

Supplementary Figure 11: Scatter plots of observed BLUEs versus predicted values for grain yield across test breeding stages, comparing models that include breeding stage effects (–YesBS) and those that exclude them (–NoBS) under analysis strategy 1 (TS-S1) and analysis strategy 2 (TS-S2). The red colored circles indicates the clusters.

Supplementary Figure 12: Scatter plots of observed BLUEs versus predicted values for plant height across test breeding stages, comparing models that include breeding stage effects (–YesBS) and those that exclude them (–NoBS) under analysis strategy 1 (TS-S1) and analysis strategy 2 (TS-S2). The red colored circles indicates the clusters

Supplementary Figure 13: Scatter plots of observed BLUEs versus predicted values for heading date across test breeding stages, comparing models that include breeding stage effects (–YesBS) and those that exclude them (–NoBS) under analysis strategy 1 (TS-S1) and analysis strategy 2 (TS-S2).

Supplementary Figure 14: Distribution of within-environment BLUEs for environments with more than one breeding stage across years for grain yield: (a) 2020, (b) 2021, (c) 2022, and (d) 2023. Different colors indicate different breeding stages. The distributions show that, in some environments, there are clear systematic differences in performance among breeding stages.

Supplementary Figure 15: Distribution of within-environment BLUEs for environments with more than one breeding stage across years for plant height: (a) 2021, (b) 2022, and (c) 2023; and for heading date: (d) 2023. Different colors indicate different breeding stages. The distributions show that, in some environments, there are clear systematic differences in performance among breeding stages.

Supplementary Figure 16: Scatter plots showing BLUEs (x-axis) versus predicted values (y-axis) for grain yield in (a) 2021 (a year with no observed breeding-stage effect) and (b) 2022 (a year with a strong breeding-stage effect) using the TS-S1-NoBS model across the initial five runs of the 5-fold cross-validation. The r-values in each subplot represent the correlation between BLUEs and predicted values (prediction ability). The presence of clustering by breeding stage (indicated by coloured points) in (b) suggests that, in the presence of a breeding-stage effect, the TS-S1-NoBS model produces BLUEs that are confounded by environmental effects associated with breeding stage, leading to an overestimation of prediction ability in each run. This pattern is not observed when the breeding-stage effect is absent or negligible, as shown in (a).

# **Supplementary tables**

Supplementary Table 1: Summary of the number of genotypes with phenotypic observations (n genotypes), genotypes with both genotypic and phenotypic data (n genotypes GP), and the number of test locations (n locations), stratified by year, trait, and breeding stage.

| **Year** | **Trait** | **Breeding stage** | **n genotypes** | **n locations** | **n genotypes GP** |
| --- | --- | --- | --- | --- | --- |
| Across | Grain yield | Across | 3972 | 28 | 1939 |
| 2020 | Grain yield | Across | 913 | 7 | 229 |
| 2021 | Grain yield | Across | 1001 | 20 | 418 |
| 2022 | Grain yield | Across | 1004 | 20 | 330 |
| 2023 | Grain yield | Across | 1002 | 18 | 976 |
| 2020 | Grain yield | WP-1 | 69 | 17 | 69 |
| 2020 | Grain yield | WP-2 | 179 | 6 | 170 |
| 2020 | Grain yield | WP-3 | 737 | 4 | 53 |
| 2021 | Grain yield | WP-1 | 69 | 17 | 69 |
| 2021 | Grain yield | WP-2 | 223 | 6 | 204 |
| 2021 | Grain yield | WP-3 | 716 | 3 | 144 |
| 2022 | Grain yield | WP-1 | 69 | 17 | 67 |
| 2022 | Grain yield | WP-2 | 201 | 6 | 138 |
| 2022 | Grain yield | WP-3 | 737 | 4 | 125 |
| 2023 | Grain yield | WP-1 | 69 | 15 | 69 |
| 2023 | Grain yield | WP-2 | 223 | 5 | 207 |
| 2023 | Grain yield | WP-3 | 716 | 3 | 698 |
| Across | Heading date | Across | 2580 | 13 | 1375 |
| 2021 | Heading date | Across | 781 | 7 | 214 |
| 2022 | Heading date | Across | 806 | 7 | 192 |
| 2023 | Heading date | Across | 1002 | 8 | 978 |
| 2021 | Heading date | WP-1 | 69 | 6 | 69 |
| 2021 | Heading date | WP-3 | 716 | 1 | 144 |
| 2022 | Heading date | WP-1 | 69 | 5 | 67 |
| 2022 | Heading date | WP-3 | 737 | 2 | 125 |
| 2023 | Heading date | WP-1 | 69 | 7 | 69 |
| 2023 | Heading date | WP-2 | 223 | 2 | 207 |
| 2023 | Heading date | WP-3 | 716 | 3 | 700 |
| Across | Plant height | Across | 2998 | 14 | 1717 |
| 2021 | Plant height | Across | 1001 | 8 | 418 |
| 2022 | Plant height | Across | 1004 | 9 | 330 |
| 2023 | Plant height | Across | 1002 | 9 | 978 |
| 2021 | Plant height | WP-1 | 69 | 7 | 69 |
| 2021 | Plant height | WP-2 | 223 | 1 | 204 |
| 2021 | Plant height | WP-3 | 716 | 2 | 144 |
| 2022 | Plant height | WP-1 | 69 | 8 | 67 |
| 2022 | Plant height | WP-2 | 201 | 3 | 138 |
| 2022 | Plant height | WP-3 | 737 | 1 | 125 |
| 2023 | Plant height | WP-1 | 69 | 8 | 69 |
| 2023 | Plant height | WP-2 | 223 | 3 | 207 |
| 2023 | Plant height | WP-3 | 716 | 2 | 700 |

Supplementary Table 2: Summary of the number of genotypes genotyped with the 7K and 25K marker chips across different years.

| **Year** | **Chip** | **Genotypes** |
| --- | --- | --- |
| 2020 | 7K | 255 |
| 2021 | 7K | 346 |
| 2022 | 7K | 263 |
| 2023 | 7K | 905 |
| 2020 | 25K | 69 |
| 2021 | 25K | 69 |
| 2022 | 25K | 69 |
| 2023 | 25K | 69 |

Supplementary Table 3: The mean and standard deviation (SD) of the Rogers’ distance between the genotypes within the breeding trial stage and between the breeding trial stages.

| **Dataset** | **Mean** | **SD** |
| --- | --- | --- |
| WP-1 | 0.36 | 0.04 |
| WP-2 | 0.36 | 0.03 |
| WP-3 | 0.36 | 0.03 |
| WP-1 & WP-2 | 0.36 | 0.03 |
| WP-1 & WP-3 | 0.37 | 0.03 |
| WP-2 & WP-3 | 0.37 | 0.03 |

Supplementary Table 4: The mean and standard deviation (SD) of the Rogers’ distance between the genotypes within the year and between the years.

| **Dataset** | **Mean** | **SD** |
| --- | --- | --- |
| 2020 | 0.37 | 0.04 |
| 2021 | 0.36 | 0.03 |
| 2022 | 0.36 | 0.03 |
| 2023 | 0.36 | 0.03 |
| 2020 & 2021 | 0.37 | 0.03 |
| 2020 & 2022 | 0.37 | 0.03 |
| 2020 & 2023 | 0.37 | 0.02 |
| 2021 & 2022 | 0.37 | 0.03 |
| 2021 & 2023 | 0.37 | 0.03 |
| 2022 & 2023 | 0.36 | 0.03 |

Supplementary Table 5: Overview of the environments removed during stage one of the analysis strategy 1 across traits and years due to repeatability lower than 0.30.

| **Year** | **Location** | **Site** | **Breeding stage** | **Trait** | **Repeatability** |
| --- | --- | --- | --- | --- | --- |
| 2020 | Gerhaus | 2020_GER | WP-1 | Grain yield | 1.23E-07 |
| 2022 | Herzogenaurach | 2022_HEZ | WP-1 | Grain yield | 8.55E-07 |
| 2022 | Lemgo | 2022_LEM | WP-1 | Grain yield | 2.20E-07 |
| 2023 | Herzogenaurach | 2023_HEZ | WP-1 | Grain yield | 2.13E-07 |
| 2023 | Thüle | 2023_THU | WP-2 | Grain yield | 1.36E-06 |
| 2023 | Gudow | 2023_GUD | WP-1 | Plant height | 9.40E-08 |

Supplementary Table 6: Mean prediction ability across years from 5-fold cross-validation (analysis strategy 1) for grain yield, plant height, and heading date, comparing models with (TS-S1-YesBS) and without (TS-S1-NoBS) breeding stage in the environment effect.

| **Trait** | **Year** | **Model** | **Mean prediction ability** | **Standard error** |
| --- | --- | --- | --- | --- |
| Grain yield | 2020 | TS-S1-NoBS | 0.54 | 0.0032 |
| Grain yield | 2021 | TS-S1-NoBS | 0.43 | 0.0029 |
| Grain yield | 2022 | TS-S1-NoBS | 0.76 | 0.0014 |
| Grain yield | 2023 | TS-S1-NoBS | 0.56 | 0.0013 |
| Grain yield | Across years | TS-S1-NoBS | 0.55 | 0.0011 |
| Grain yield | 2020 | TS-S1-YesBS | 0.53 | 0.0029 |
| Grain yield | 2021 | TS-S1-YesBS | 0.45 | 0.0029 |
| Grain yield | 2022 | TS-S1-YesBS | 0.69 | 0.0018 |
| Grain yield | 2023 | TS-S1-YesBS | 0.54 | 0.0013 |
| Grain yield | Across years | TS-S1-YesBS | 0.58 | 0.0010 |
| Heading date | 2021 | TS-S1-NoBS | 0.24 | 0.0045 |
| Heading date | 2022 | TS-S1-NoBS | 0.66 | 0.0022 |
| Heading date | 2023 | TS-S1-NoBS | 0.64 | 0.0012 |
| Heading date | Across years | TS-S1-NoBS | 0.64 | 0.0008 |
| Heading date | 2021 | TS-S1-YesBS | 0.24 | 0.0045 |
| Heading date | 2022 | TS-S1-YesBS | NA | NA |
| Heading date | 2023 | TS-S1-YesBS | 0.62 | 0.0013 |
| Heading date | Across years | TS-S1-YesBS | 0.63 | 0.0010 |
| Plant height | 2021 | TS-S1-NoBS | 0.52 | 0.0025 |
| Plant height | 2022 | TS-S1-NoBS | 0.64 | 0.0019 |
| Plant height | 2023 | TS-S1-NoBS | 0.58 | 0.0015 |
| Plant height | Across years | TS-S1-NoBS | 0.63 | 0.0010 |
| Plant height | 2021 | TS-S1-YesBS | 0.52 | 0.0026 |
| Plant height | 2022 | TS-S1-YesBS | 0.37 | 0.0030 |
| Plant height | 2023 | TS-S1-YesBS | 0.39 | 0.0020 |
| Plant height | Across years | TS-S1-YesBS | 0.51 | 0.0012 |

Supplementary Table 7: Mean prediction ability across years from 5-fold cross-validation (analysis strategy 2) for grain yield, plant height, and heading date, comparing models with (TS-S2-YesBS) and without (TS-S2-NoBS) breeding stage effect.

| **Trait** | **Year** | **Model** | **Mean prediction ability** | **Standard error** |
| --- | --- | --- | --- | --- |
| Grain yield | 2020 | TS-S2-NoBS | 0.47 | 0.0035 |
| Grain yield | 2021 | TS-S2-NoBS | 0.50 | 0.0028 |
| Grain yield | 2022 | TS-S2-NoBS | 0.60 | 0.0024 |
| Grain yield | 2023 | TS-S2-NoBS | 0.57 | 0.0011 |
| Grain yield | Across years | TS-S2-NoBS | 0.59 | 0.0007 |
| Grain yield | 2020 | TS-S2-YesBS | 0.48 | 0.0031 |
| Grain yield | 2021 | TS-S2-YesBS | 0.47 | 0.0029 |
| Grain yield | 2022 | TS-S2-YesBS | 0.64 | 0.0018 |
| Grain yield | 2023 | TS-S2-YesBS | 0.55 | 0.0014 |
| Grain yield | Across years | TS-S2-YesBS | 0.60 | 0.0007 |
| Heading date | 2021 | TS-S2-NoBS | 0.24 | 0.0041 |
| Heading date | 2022 | TS-S2-NoBS | 0.66 | 0.0023 |
| Heading date | 2023 | TS-S2-NoBS | 0.64 | 0.0014 |
| Heading date | Across years | TS-S2-NoBS | 0.66 | 0.0012 |
| Heading date | 2021 | TS-S2-YesBS | 0.21 | 0.0052 |
| Heading date | 2022 | TS-S2-YesBS | NA | NA |
| Heading date | 2023 | TS-S2-YesBS | 0.63 | 0.0013 |
| Heading date | Across years | TS-S2-YesBS | 0.63 | 0.0010 |
| Plant height | 2021 | TS-S2-NoBS | 0.50 | 0.0032 |
| Plant height | 2022 | TS-S2-NoBS | 0.51 | 0.0030 |
| Plant height | 2023 | TS-S2-NoBS | 0.53 | 0.0016 |
| Plant height | Across years | TS-S2-NoBS | 0.51 | 0.0011 |
| Plant height | 2021 | TS-S2-YesBS | 0.51 | 0.0031 |
| Plant height | 2022 | TS-S2-YesBS | 0.45 | 0.0031 |
| Plant height | 2023 | TS-S2-YesBS | 0.41 | 0.0013 |
| Plant height | Across years | TS-S2-YesBS | 0.52 | 0.0012 |

Supplementary Table 8: Prediction ability across years (Test set) from leave-one-year-out cross-validation (analysis strategy 1) for grain yield, plant height, and heading date, comparing models with (TS-S1-YesBS) and without (TS-S1-NoBS) breeding stage in the environment effect.

| **Trait** | **Test set** | **Training set** | **Test set size** | **Training set size** | **Prediction ability** | **Model** |
| --- | --- | --- | --- | --- | --- | --- |
| Grain yield | 2020 | 2021_2022_2023 | 229 | 1710 | 0.26 | TS-S1-NoBS |
| Grain yield | 2021 | 2020_2022_2023 | 418 | 1521 | -0.07 | TS-S1-NoBS |
| Grain yield | 2022 | 2020_2021_2023 | 330 | 1609 | -0.14 | TS-S1-NoBS |
| Grain yield | 2023 | 2020_2021_2022 | 976 | 963 | 0.04 | TS-S1-NoBS |
| Grain yield | 2020 | 2021_2022_2023 | 229 | 1710 | 0.36 | TS-S1-YesBS |
| Grain yield | 2021 | 2020_2022_2023 | 418 | 1521 | 0.36 | TS-S1-YesBS |
| Grain yield | 2022 | 2020_2021_2023 | 330 | 1609 | 0.34 | TS-S1-YesBS |
| Grain yield | 2023 | 2020_2021_2022 | 976 | 963 | 0.33 | TS-S1-YesBS |
| Plant height | 2021 | 2022_2023 | 418 | 1299 | 0.3 | TS-S1-NoBS |
| Plant height | 2022 | 2021_2023 | 330 | 1387 | 0.51 | TS-S1-NoBS |
| Plant height | 2023 | 2021_2022 | 978 | 739 | 0.25 | TS-S1-NoBS |
| Plant height | 2021 | 2022_2023 | 418 | 1299 | 0.39 | TS-S1-YesBS |
| Plant height | 2022 | 2021_2023 | 330 | 1387 | 0.56 | TS-S1-YesBS |
| Plant height | 2023 | 2021_2022 | 978 | 739 | 0.39 | TS-S1-YesBS |
| Heading date | 2021 | 2022_2023 | 214 | 1161 | 0.14 | TS-S1-NoBS |
| Heading date | 2022 | 2021_2023 | 192 | 1183 | 0.65 | TS-S1-NoBS |
| Heading date | 2023 | 2021_2022 | 978 | 397 | 0.45 | TS-S1-NoBS |
| Heading date | 2021 | 2022_2023 | 214 | 1161 | 0.18 | TS-S1-YesBS |
| Heading date | 2022 | 2021_2023 | 192 | 1183 | 0.65 | TS-S1-YesBS |
| Heading date | 2023 | 2021_2022 | 978 | 397 | 0.42 | TS-S1-YesBS |

Supplementary Table 9: Prediction ability across years (Test set) from leave-one-year-out cross-validation (analysis strategy 2) for grain yield, plant height, and heading date, comparing models with (TS-S2-YesBS) and without (TS-S2-NoBS) breeding stage effect.

| **Trait** | **Test set** | **Training set** | **Test set size** | **Training set size** | **Prediction ability** | **Model** |
| --- | --- | --- | --- | --- | --- | --- |
| Grain yield | 2020 | 2021_2022_2023 | 229 | 1709 | 0.4 | TS-S2-NoBS |
| Grain yield | 2021 | 2020_2022_2023 | 418 | 1520 | 0.4 | TS-S2-NoBS |
| Grain yield | 2022 | 2020_2021_2023 | 330 | 1608 | 0.25 | TS-S2-NoBS |
| Grain yield | 2023 | 2020_2021_2022 | 976 | 963 | 0.18 | TS-S2-NoBS |
| Grain yield | 2020 | 2021_2022_2023 | 229 | 1710 | 0.46 | TS-S2-YesBS |
| Grain yield | 2021 | 2020_2022_2023 | 418 | 1521 | 0.53 | TS-S2-YesBS |
| Grain yield | 2022 | 2020_2021_2023 | 330 | 1609 | 0.19 | TS-S2-YesBS |
| Grain yield | 2023 | 2020_2021_2022 | 976 | 963 | 0.41 | TS-S2-YesBS |
| Plant height | 2021 | 2022_2023 | 418 | 1299 | 0.21 | TS-S2-NoBS |
| Plant height | 2022 | 2021_2023 | 330 | 1387 | 0.25 | TS-S2-NoBS |
| Plant height | 2023 | 2021_2022 | 978 | 739 | 0.11 | TS-S2-NoBS |
| Plant height | 2021 | 2022_2023 | 418 | 1299 | 0.37 | TS-S2-YesBS |
| Plant height | 2022 | 2021_2023 | 330 | 1387 | 0.46 | TS-S2-YesBS |
| Plant height | 2023 | 2021_2022 | 978 | 739 | 0.29 | TS-S2-YesBS |
| Heading date | 2021 | 2022_2023 | 214 | 1161 | 0.07 | TS-S2-NoBS |
| Heading date | 2022 | 2021_2023 | 192 | 1183 | 0.67 | TS-S2-NoBS |
| Heading date | 2023 | 2021_2022 | 978 | 397 | 0.33 | TS-S2-NoBS |
| Heading date | 2021 | 2022_2023 | 214 | 1161 | 0.11 | TS-S2-YesBS |
| Heading date | 2022 | 2021_2023 | 192 | 1183 | 0.66 | TS-S2-YesBS |
| Heading date | 2023 | 2021_2022 | 978 | 397 | 0.32 | TS-S2-YesBS |

Supplementary Table 10: Example table showing the proportion of genotypes from each breeding stage relative to the total number of genotypes in 2022. N represents the number of genotypes belonging to each breeding stage.

| **Year** | **Population** | **Breeding stage** | **Total genotypes** | **N** | **Proportion (%)** |
| --- | --- | --- | --- | --- | --- |
| 2022 | Base population | Multi | 330 | 3 | 0.91 |
| 2022 | Base population | WP-1 | 330 | 64 | 19.39 |
| 2022 | Base population | WP-2 | 330 | 138 | 41.82 |
| 2022 | Base population | WP-3 | 330 | 125 | 37.88 |

Supplementary Table 11: Example table showing the mean proportion of genotypes from each breeding stage in each fold across 50 runs of 5-fold cross-validation, relative to the total number of genotypes in 2022. This table shows that, in each fold, the average proportion of genotypes from each breeding stage is similar to the proportions observed in the base population (Supplementary Table 10). Therefore, if systematic clustering by breeding stage is present in the base population, it will also be reflected in each training and test set in every run of the 5-fold cross-validation, potentially leading to unreliable estimates of prediction ability.

| **Year** | **Fold** | **Breeding stage** | **Mean proportion (%)** | **Standard deviation** |
| --- | --- | --- | --- | --- |
| 2022 | Fold1 | Multi | 1.84 | 0.64 |
| 2022 | Fold1 | WP-1 | 19.52 | 3.30 |
| 2022 | Fold1 | WP-2 | 42.13 | 4.60 |
| 2022 | Fold1 | WP-3 | 37.65 | 3.06 |
| 2022 | Fold2 | Multi | 1.94 | 0.68 |
| 2022 | Fold2 | WP-1 | 19.31 | 3.16 |
| 2022 | Fold2 | WP-2 | 41.54 | 4.36 |
| 2022 | Fold2 | WP-3 | 38.03 | 2.94 |
| 2022 | Fold3 | Multi | 2.05 | 0.75 |
| 2022 | Fold3 | WP-1 | 19.08 | 3.54 |
| 2022 | Fold3 | WP-2 | 42.12 | 4.05 |
| 2022 | Fold3 | WP-3 | 37.97 | 2.72 |
| 2022 | Fold4 | Multi | 1.94 | 0.82 |
| 2022 | Fold4 | WP-1 | 19.74 | 3.71 |
| 2022 | Fold4 | WP-2 | 41.16 | 4.44 |
| 2022 | Fold4 | WP-3 | 38.13 | 2.62 |
| 2022 | Fold5 | Multi | 1.73 | 0.56 |
| 2022 | Fold5 | WP-1 | 19.32 | 3.63 |
| 2022 | Fold5 | WP-2 | 42.11 | 4.46 |
| 2022 | Fold5 | WP-3 | 37.64 | 2.54 |
